# Knowledge, Attitudes, Practices, and Vaccination Willingness Toward Mpox (Monkeypox) Among Chinese Medical Students: Cross-Sectional Study

**DOI:** 10.2196/86981

**Published:** 2026-02-06

**Authors:** Yang Liu, Yuehui Jia, Honglong Li, Jie Ge, Yunfeng Han, Zhiping Xie, Jiaxin Chen

**Affiliations:** 1 Scientific Research Department, Qiqihar Medical University, Qiqihar, Heilongjiang, China; 2 School of Public Health, Qiqihar Medical University, 333 Bukui Street, Jianhua District, Qiqihar, Heilongjiang, 161000, China, 86 452-2663409, 86 452-2663755; 3 School of Mental Health, Qiqihar Medical University, Qiqihar, Heilongjiang, China

**Keywords:** monkeypox, mpox, mpox vaccination, vaccine hesitancy, determinants, knowledge, attitude, practice

## Abstract

**Background:**

Mpox (monkeypox) remains a global public health threat. However, data on mpox-related knowledge, attitudes, and practices (KAP) and vaccination willingness among Chinese medical students, who are key future health care practitioners, remain lacking.

**Objective:**

This study aimed to investigate systematically the KAP and mpox vaccination willingness of Chinese medical students and identify the factors influencing their vaccination decisions.

**Methods:**

A nationwide cross-sectional survey was conducted from November 2023 to March 2024. An anonymous self-designed questionnaire was used to assess basic information, KAP toward mpox, vaccination-related behaviors, and willingness. Categorical data were presented as frequency (constituent ratio). The normality of continuous variables was assessed using the Kolmogorov-Smirnov test. Continuous variables that did not conform to a normal distribution were presented as median (IQR). Data were analyzed using the chi-square test, 2-tailed *t* test, ANOVA, Kruskal-Wallis *H* test, and multinomial logistic regression.

**Results:**

Among the 4098 participants, 84.63% (n=3468) accepted mpox vaccination. The median scores of KAP toward mpox were 43 (IQR 33-50), 33 (IQR 32-36), and 20 (IQR 19-24), respectively, with a median score of 73 (IQR 68-79) for vaccination-related practices. Multinomial logistic regression showed that factors associated with vaccination hesitancy (vs acceptance) included male individuals (odds ratio [OR] 1.416, 95% CI 1.158‐1.732), being an only child (OR 1.340, 95% CI 1.098‐1.635), no history of COVID-19 in family or friends (OR 1.520, 95% CI 1.161‐1.991), no influenza vaccination (OR 1.429, 95% CI 1.146‐1.783), and low mpox knowledge (OR 0.948, 95% CI 0.941‐0.955). Factors associated with vaccination rejection (vs acceptance) included male sex (OR 1.641, 95% CI 1.003‐2.686), high academic grade (OR 1.442, 95% CI 1.154‐1.802), family or friends working on COVID-19 frontlines (OR 2.243, 95% CI 1.337‐3.764), no internship experience (OR 2.049, 95% CI 1.076‐3.901), presence of organic diseases (OR 3.733, 95% CI 1.778‐7.838), and low mpox knowledge (OR 0.954, 95% CI 0.938‐0.971). Good self-reported health status was a protective factor against refusal (OR 0.748, 95% CI 0.580‐0.965).

**Conclusions:**

The high willingness to receive mpox vaccination among Chinese medical students and its determinants, as identified in this study, carry clear implications for both education and policy. These findings inform the design of targeted health education programs for students and guide the development of evidence-based prevention strategies on campuses during public health emergencies.

## Introduction

Monkeypox (mpox) is a zoonotic infectious disease caused by the mpox virus (MPXV) [1], with universal susceptibility in the human population. Its clinical manifestations are similar to those of smallpox, including fever, rash, and malaise. A key difference from smallpox is the higher prevalence of lymphadenopathy in mpox cases [2]. The first human case of mpox was identified in the Democratic Republic of the Congo (then known as Zaire) in 1970, after which the virus was primarily endemic in west and central Africa [3]. Since May 2022, a large-scale mpox outbreak has re-emerged globally [4]. In July 2024, a more virulent new strain of MPXV (clade Ib) began spreading rapidly in the Democratic Republic of the Congo. Initially prevalent among sex workers, this strain has now spread to other populations [5,6]. As of now, a total of 162,785 mpox cases and 424 deaths have been reported across 140 countries worldwide [7]. In China, 767 infections have been documented in 2025 [8]. The mpox epidemic may have catastrophic consequences for public health, socioeconomic factors, and the entire health care system. On July 23, 2022, the World Health Organization declared the mpox epidemic a “Public Health Emergency of International Concern” [9], and reaffirmed this declaration on August 14, 2024 [10]. On September 15, 2023, the National Health Commission of the People’s Republic of China classified mpox as a category B infectious disease for management, which took effect on September 20, 2023 [11]. In accordance with the Law of the People’s Republic of China on the Prevention and Control of Infectious Diseases, category B infectious diseases are subject to strict management [12].

Public health measures to prevent mpox transmission include enhancing public awareness of the disease; however, vaccination remains the core measure. Previous studies have demonstrated that smallpox vaccination provides at least 85% effectiveness in preventing MPXV infection [13]. The World Health Organization recommends vaccination for priority populations, but vaccine hesitancy is widespread globally [14-17]. Previous experience with COVID-19 vaccine hesitancy highlights the need for targeted research on vaccination willingness toward mpox [18,19].

Medical students represent a crucial demographic in epidemic preparedness. As future frontline health care professionals and influential health communicators, their knowledge, attitudes, practices (KAP), and willingness to be vaccinated directly will impact clinical response capacity, the effectiveness of community health education, and public health messaging during outbreaks. While prior research indicates a generally high willingness to receive the mpox vaccine among Chinese health care workers [17], data regarding medical students’ KAP related to mpox and their willingness to receive the vaccine are lacking. This study used a cross-sectional online survey to investigate the mpox-related KAP of Chinese medical students and their willingness to be vaccinated against mpox, as well as the factors influencing their vaccination willingness. These factors may serve as key targets for controlling the current mpox outbreak and rapidly responding to future epidemic outbreaks. Therefore, this study provides a basis for policy decisions regarding targeted educational interventions and vaccination strategies, thereby enhancing preparedness for potential mpox outbreaks.

## Methods

### Study Design

A nationwide population-based cross-sectional survey was conducted from November 2023 to March 2024, and the snowball sampling method was adopted to recruit participants. Data were collected through an anonymous, self-administered questionnaire designed to assess Chinese medical students’ demographic characteristics and KAP related to mpox, as well as vaccination-related behaviors and willingness. The study follows the STROBE (Strengthening the Reporting of Observational Studies in Epidemiology) guidelines (Checklist 1).

### Study Objects

The study population met the following inclusion criteria: (1) full-time medical students in mainland China, (2) currently enrolled in a degree program, and (3) willing to complete a questionnaire. A total of 4320 students participated in the survey, among whom 4098 valid questionnaires were collected, with an effective response rate of 94.86%.

### Survey Methods

An anonymous online survey was conducted via the Wenjuanxing platform, a professional online survey platform in China, and snowball sampling was used to recruit participants. All the questionnaires were edited, and an electronic 2D code for the survey was generated through the Wenjuanxing online platform. The process was initiated by the research team contacting academic advisors and student leaders from various medical schools. These initial contacts were provided with a standardized recruitment package, which included a brief study description, eligibility criteria, and the unique 2D code link to the questionnaire. The initial contacts distributed the recruitment package to potential participants via QQ (Tencent), a popular instant messaging software in China, and WeChat (Tencent), a multifunctional social media and messaging app. Specifically, dissemination occurred through a combination of private messages, academic group chats, and institutional social media channels. Additionally, participants were encouraged to assist in recruiting other study individuals by sharing the electronic 2D code of the questionnaire.

Students scanned the 2D code of the questionnaire using electronic devices, participated in the survey voluntarily, and completed the questionnaire. All items in the questionnaire were set as mandatory to ensure data integrity. Each IP address was restricted to submitting the questionnaire only once to prevent duplicate submissions. Furthermore, the time spent completing the questionnaire was automatically monitored by the Wenjuanxing platform; responses were considered invalid if the completion time was less than 180 seconds.

### Survey Instruments

A self-designed questionnaire was used to investigate medical students’ KAP regarding mpox, their willingness to receive mpox vaccination, and the influencing factors thereof. The questionnaire was developed on the basis of the *Technical Guidelines for Mpox Prevention and Control* (2022 version) [20]. To ensure its content validity, the initial draft was reviewed by 4 experts in public health and health education, after which it was revised. Subsequently, a pilot survey was conducted to test the clarity and feasibility of the instrument, leading to the final version used in the study.

The questionnaire consisted of 6 sections with a total of 81 items, including basic information (20 items), knowledge (23 items), attitude (11 items), practice (5 items), vaccination-related behaviors (21 items), and vaccination willingness (1 item). For the knowledge section, 2 points were awarded for each correct answer to single-choice questions, and no points for incorrect answers; for multiple-choice questions, 1 point was given for each correctly selected option. The attitude, practice, vaccination-related behaviors, and vaccination willingness sections were scored using a 5-point Likert scale, with response options of “Strongly disagree,” “Disagree,” “Neutral,” “Agree,” and “Strongly agree,” corresponding to scores of 1, 2, 3, 4, and 5, respectively. Respondents were required to rate each question. The total score of the questionnaire was 245 points. The maximum scores for the 4 sections, knowledge, attitude, practice, and vaccination-related behaviors, were 60, 55, 25, and 105 points, respectively. The Cronbach α coefficient of the questionnaire was 0.915, indicating good reliability of the questionnaire.

### Statistical Methods

SPSS (Statistical Package for the Social Sciences) 24.0 software (IBM Corp) was used for data analysis. Categorical data were presented as frequency (constituent ratio), and comparisons between groups were performed using the chi-square test or the Fisher exact test. The Kolmogorov-Smirnov test was used to test the normality of continuous variables. Continuous variables that conformed to a normal distribution were expressed as mean (SD), and comparisons between groups were conducted using the 2-tailed *t* test or ANOVA. Continuous variables that did not conform to a normal distribution were presented as median [IQR], and comparisons between groups were performed using the Kruskal-Wallis *H* test. Multinomial logistic regression was performed to identify the factors correlated with the willingness to receive the mpox vaccine. The willingness to receive the mpox vaccine was taken as the dependent variable, which included 3 categories: acceptance, hesitancy, and rejection. In the multinomial logistic regression model, the status of the acceptance group served as the reference category for the dependent variable. Variables with *P*<.10 in univariable analyses were considered as independent variables in the multinomial logistic regression model. A stepwise selection method (entry and removal criteria: *P*=.05) was used for predictor selection. The risks of the factors were displayed as odds ratios (OR) and the corresponding 95% CIs. The *P* value <.05 (2-sided) was considered statistically significant.

### Ethical Considerations

This study was approved by the ethics committee of Qiqihar Medical University (approval: 202371). This study adhered to the Declaration of Helsinki. Participants in this research participated voluntarily and were conducted anonymously through an online platform. The questionnaire did not include any identifying or sensitive content. All participants provided online informed consent. Participants did not receive any financial or nonfinancial compensation for their participation.

## Results

### Demographic Characteristics of Medical Students and Their Willingness to Receive Mpox Vaccination

A total of 4098 participants were included in the study, which included 1739 male participants and 2359 female participants. Among them, 3468 individuals accepted mpox vaccination, accounting for 84.63%; 550 individuals expressed hesitancy, representing 13.42%; and 80 individuals refused, making up 1.95%.

The willingness of medical students to receive mpox vaccination showed significant differences between sex (*χ*^2^_2_=42.5; *P*<.001), grade (*χ*^2^_8_=23.8; *P*=.003), being an only child (*χ*^2^_2_=8.2; *P*=.02), the reason for choosing medicine (*χ*^2^_2_=11.1; *P*=.004), having parents or relatives who worked in the frontline during the COVID-19 outbreak (*χ*^2^_2_=10.0; *P*=.007), self-health status (*χ*^2^_8_=59.4; *P*<.001), chronic disease (*χ*^2^_2_=14.1; *P*<.001), allergic constitution (*χ*^2^_2_=8.6; *P*=.01), organic disease (*χ*^2^_2_=26.0; *P*<.001), having family members or friends who had experienced COVID-19 (*χ*^2^_2_=40.6; *P*<.001), having received the influenza vaccination (*χ*^2^_2_=24.2; *P*<.001), having received the COVID-19 vaccination (*χ*^2^_2_=47.6; *P*<.001), and having considered the reason for receiving COVID-19 vaccination (*χ*^2^_2_=35.4; *P*<.001). The details are presented in Multimedia Appendix 1.

### KAP Status Regarding Mpox Among Medical Students and Their Willingness to Receive Mpox Vaccination

**Figure 1.**
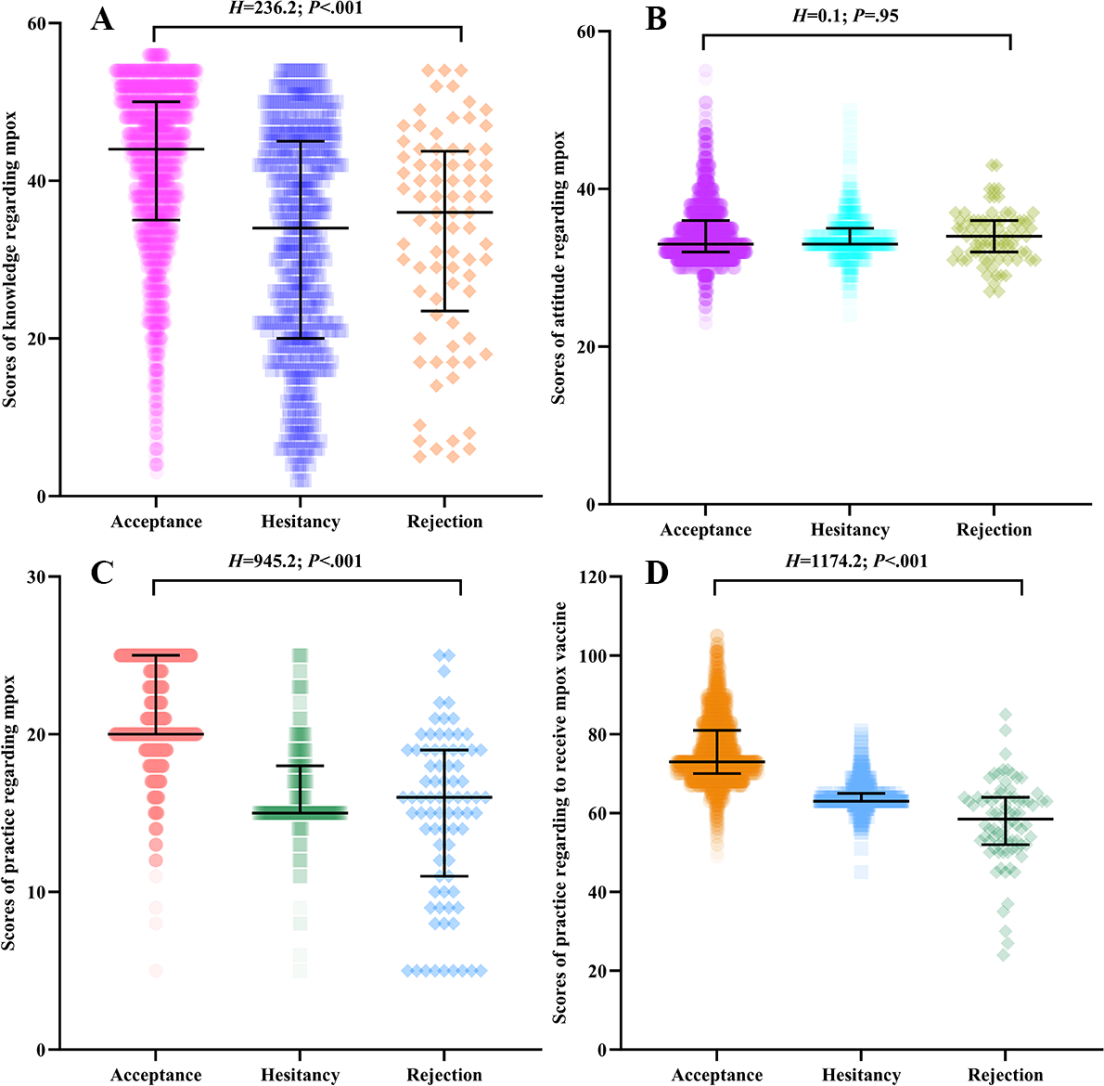
KAP status regarding monkeypox (mpox) and practice status regarding receiving mpox vaccination among Chinese medical students: (A) scores of knowledge regarding mpox; (B) scores of attitude regarding mpox; (C) scores of practice regarding mpox; and (D) scores of practice regarding receiving mpox vaccine. The numbers are represented by median (IQR). KAP: knowledge, attitude, and practice.

The maximum scores for the 4 sections—knowledge, attitude, practice, and vaccination-related behaviors—were 60, 55, 25, and 105 points, respectively. The median scores for KAP status regarding mpox among medical students were 43 (IQR 33-50), 33 (IQR 32-36), and 20 (IQR 19-24), respectively. The median for practice status regarding receiving the mpox vaccine among medical students was 73 (IQR 68-79). A significant difference was found between willingness to receive mpox vaccination and knowledge level regarding mpox (*H*=236.2; *P*<.001), practice status regarding mpox (*H*=945.2; *P*<.001), and practice status regarding receiving mpox vaccine (*H*=1174.2; *P*<.001). Table 1 outlines the details, and Figure 1 illustrates the results.

**Table 1.** Knowledge, attitude, and practice (KAP) status regarding monkeypox (mpox) among Chinese medical students and its association with their willingness to receive mpox vaccination.

Variables	All participants, median (IQR)	Willingness to receive mpox vaccine, median (IQR)			*H* value^a^	*P* value
		Acceptance	Hesitancy	Rejection		
Knowledge status regarding mpox	43 (33-50)	44 (35-50)	34 (20-45)	36 (24-43.5)	236.2	<.001^b^
Attitude status regarding mpox	33 (32-36)	33 (32-36)	33 (33-35)	34 (32-36)	0.1	.95
Practice status regarding mpox	20 (19-24)	20 (20-25)	15 (15-18)	16 (11-19)	945.2	<.001^b^
Practice status regarding receiving mpox vaccine	73 (68-79)	73 (70-81)	63 (63-65)	58.5 (52-64)	1174.2	<.001^b^

a Kruskal-Wallis *H* test.

b *P*<.05 and the difference was statistically significant.

### Multinomial Logistic Regression for Identification of the Factors Correlated With the Willingness to Receive Mpox Vaccination

**Figure 2.**
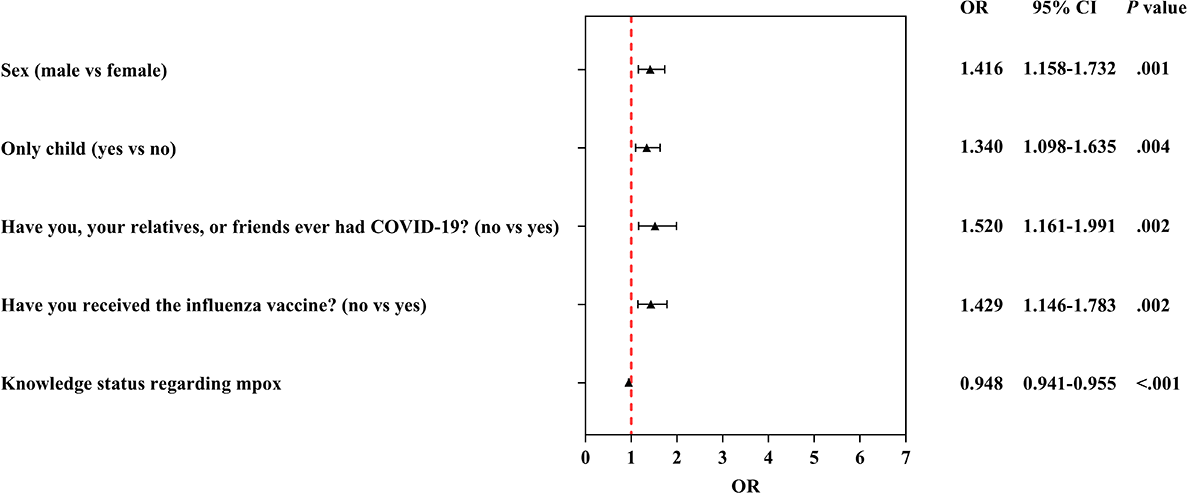
Multinomial logistic regression analysis of the determinants significantly associated with hesitancy to receive monkeypox (mpox) vaccination among Chinese medical students. OR: odds ratio.

Compared with the acceptance group, factors such as sex, only-child status, history of COVID-19 infection among students or their relatives and friends, prior influenza vaccination, and knowledge level regarding mpox were significantly correlated with hesitancy to receive the mpox vaccine. Male sex was a risk factor for vaccine hesitancy, with a 1.416-fold risk than female sex (OR 1.416, 95% CI 1.158-1.732). Compared with their counterpart groups, participants who were the only child in their family (OR 1.340, 95% CI 1.098‐1.635), those who had never contracted COVID-19 with their relatives or friends (OR 1.520, 95% CI 1.161-1.991), those who had not received the influenza vaccine (OR 1.429, 95% CI 1.146‐1.783), and those with low knowledge level regarding mpox (OR 0.948, 95% CI 0.941‐0.955) displayed an increased hesitancy to receive mpox vaccination. Table 2 outlines the details, and Figure 2 illustrates the results.

**Table 2.** Multivariable analysis of factors correlated with monkeypox (mpox) vaccination willingness among medical students in China^a^.

Variables	β	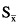	Wald chi-square (*df*)	*P* value	OR^b^ (95% CI)	
Hesitancy						
Sex (reference: female)						
Male	0.3481	0.1028	11.5 (1)	.001	1.416 (1.158‐1.732)	
Only child (reference: no)						
Yes	0.2928	0.1016	8.3 (1)	.004	1.340 (1.098‐1.635)	
Have you, your relatives, or friends ever had COVID-19? (reference: yes)						
No	0.4190	0.1376	9.3 (1)	.002	1.520 (1.161‐1.991)	
Have you received the influenza vaccine? (reference: yes)						
No	0.3573	0.1127	10.1 (1)	.002	1.429 (1.146‐1.783)	
Knowledge status regarding mpox	−0.0537	0.0039	194.5 (1)	<.001	0.948 (0.941‐0.955)	
Rejection						
Sex (reference: female)						
Male	0.4955	0.2513	3.9 (1)	.049	1.641 (1.003‐2.686)	
Grade	0.3662	0.1137	10.4 (1)	.001	1.442 (1.154‐1.802)	
Do your parents or relatives engage in frontline work during the COVID-19 outbreak? (reference: no)						
Yes	0.8079	0.2640	9.4 (1)	.002	2.243 (1.337‐3.764)	
Do you have internship experience? (reference: yes)						
No	0.7174	0.3285	4.8 (1)	.03	2.049 (1.076‐3.901)	
Self-health status	−0.2898	0.1298	5.0 (1)	.03	0.748 (0.580‐0.965)	
Organic disease (reference: no)						
Yes	1.3171	0.3785	12.1 (1)	.001	3.733 (1.778‐7.838)	
Knowledge status regarding mpox	−0.0467	0.0090	27.3 (1)	<.001	0.954 (0.938‐0.971)	

a The willingness to receive the mpox vaccine was used as the dependent variable, which included 3 categories: acceptance, hesitancy, and rejection. In the multinomial logistic regression model, the status of the acceptance group served as the reference category for the dependent variable.

b OR: odds ratio.

**Figure 3.**
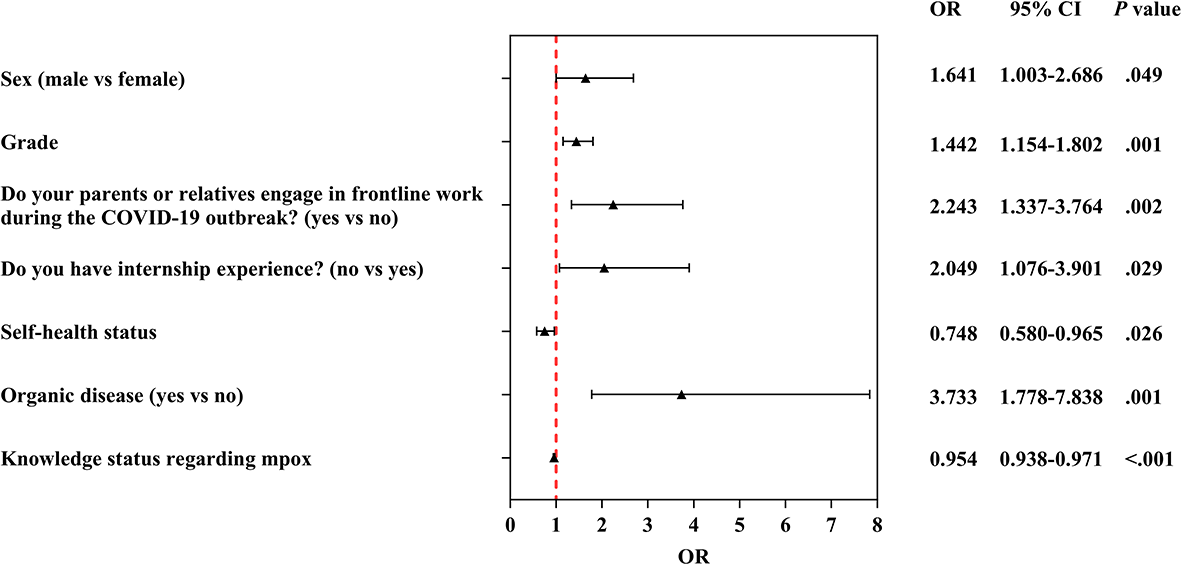
Multinomial logistic regression analysis of the determinants significantly associated with rejection to receive monkeypox (mpox) vaccination among Chinese medical students. OR: odds ratio.

Compared with the acceptance group, factors such as sex, grade, parents’ or relatives’ engagement in frontline work during the COVID-19 outbreak, internship experience, self-reported health status, presence of organic disease, and knowledge level regarding mpox were significantly correlated with rejection of mpox vaccination. Male sex was the risk factor for rejection status, with a 1.641-fold risk compared to the female sex (OR 1.641, 95% CI 1.003‐2.686). Grade was the risk factor for rejection status with a 1.442-fold risk as grade increased (OR 1.442, 95% CI 1.154‐1.802). Self-health status was the protective factor for rejection status as the health scores increased (OR 0.748, 95% CI 0.580‐0.965). Compared with their counterpart groups, participants whose parents or relatives engaged in frontline work during the COVID-19 outbreak (OR 2.243, 95% CI 1.337‐3.764), those who had no internship experience (OR 2.049, 95% CI=1.076‐3.901), those who had organic disease (OR 3.733, 95% CI 1.778‐7.838), and those with low knowledge level regarding mpox (OR 0.954, 95% CI 0.938‐0.971) displayed an increased rejection to receive mpox vaccination. Table 2 outlines the details, and Figure 3 visualizes the results.

## Discussion

### Principal Findings

This study conducted a nationwide cross-sectional online survey, targeting 4098 full-time medical students in mainland China to investigate their KAP regarding mpox and their willingness to receive the mpox vaccination systematically. As the first study to focus on Chinese medical students, a group poised to become core public health practitioners, this research fills a critical gap in domestic data on mpox-related health literacy and vaccine acceptance. The findings reveal high overall vaccine acceptance among medical students, alongside clear demographic, health-related, and KAP-associated factors influencing vaccine hesitancy and rejection. These results are not only timely, given the ongoing global mpox epidemic (with 162,785 cases and 424 deaths across 140 countries) and China’s classification of mpox as a class B notifiable disease, but also provide actionable evidence for formulating targeted educational interventions and vaccine distribution strategies. By identifying modifiable determinants of vaccine willingness, this study supports enhanced preparedness for potential future mpox outbreaks and strengthens the role of medical students as frontline communicators of public health knowledge.

The results of this study showed that 84.63% of Chinese medical students were willing to accept mpox vaccination, with 13.42% expressing hesitancy and only 1.95% refusing. This acceptance rate is remarkably higher than that reported in most studies on health care–related populations or college students. For example, a study in the Democratic Republic of the Congo involving health care workers found that the willingness rate to receive vaccines was 61% [21]; in a Saudi Arabian study, 52.7% of health care workers were willing to receive vaccines [22]; in Pakistan, the vaccine acceptance rate among college students was 67.7% [23]; while a meta-analysis conducted by Yappalparvi et al [24] showed that only 58.6% of college students were willing to receive the mpox vaccine. Another cross-sectional multinational study targeting health care providers found that the acceptability of the mpox vaccine was just over half of the participants (54.5%) [25]. Notably, the acceptance rate in our study is slightly lower than the 90.1% reported for Chinese health care workers in a 2023 systematic review by Lounis and Riad [17] but remains among the highest globally. The high vaccine acceptance among Chinese medical students can be attributed to three key factors. First, their professional medical background enables them to gain a deeper understanding of the pathogenesis of mpox, transmission risks, and the 85% protective efficacy of smallpox vaccines against mpox [13]. Furthermore, they are more capable of comprehending the high-efficiency protective mechanism of mRNA vaccines developed by scholars, which is achieved through the synergistic activation of “humoral immunity + cellular immunity” [26,27]. This knowledge reserve mitigates doubts arising from information asymmetry. Second, differences in the policy environment are equally critical. China’s strict category B management of mpox, coupled with targeted health education conducted via official guidelines, such as *Monkeypox Prevention and Control Technology* (2022 Edition) [20], has established a clear public health narrative regarding the necessity of vaccines. In addition, the experience accumulated from large-scale COVID-19 vaccination has led to a remarkably higher recognition of vaccines as public health intervention tools among Chinese medical students [28,29]. The marginal gap between medical students and practicing health care workers likely reflects differences in direct clinical exposure risk; health care workers face immediate occupational exposure, while medical students’ risk perception is more knowledge-based than experiential.

Multivariate logistic regression identified five independent factors associated with vaccine hesitancy: male sex, being an only child, no personal or familial COVID-19 history, no influenza vaccine uptake, and low mpox knowledge levels. Male participants were 1.416-fold more likely to be hesitant than female participants (OR 1.416, 95% CI 1.158‐1.732). This result is consistent with the findings of a study on COVID-19 vaccines among Saudi university students, where male individuals often exhibit low health-seeking behavior and a great tendency to downplay infectious disease risks [30]. The observed association in our study may reflect a similar pattern of risk underestimation, which could be a particularly relevant factor in decision-making regarding nonhighly lethal infectious diseases such as mpox. Only children had a 34.0% higher hesitancy risk (OR 1.340, 95% CI 1.098‐1.635). This unique finding is closely associated with the characteristics of family structures shaped by the long-term impact of China’s family planning policy. Only children often face greater parental caution regarding medical interventions, and family decision-making may prioritize avoiding potential, even rare, vaccine risks over mpox prevention. Furthermore, only children may have less exposure to collective health narratives compared with those with siblings, reducing perceived personal responsibility for vaccination. Participants without a history of COVID-19 in themselves or relatives were 1.520-fold more hesitant (OR 1.520, 95% CI 1.161‐1.991). A study in Arab countries also found that experiencing COVID-19 firsthand or through family members likely reinforces awareness of infectious disease severity and the value of preventive measures [31]. By contrast, those without such experience may perceive mpox as a “distant threat,” weakening motivation to accept vaccination. Nonrecipients of influenza vaccines had a 42.9% higher hesitancy risk (OR 1.429, 95% CI 1.146‐1.783). Influenza vaccine uptake reflects established trust in routine immunization. Nonrecipients may hold persistent doubts about vaccine safety or effectiveness, attitudes that generalize to novel vaccines such as mpox. This result suggests that routine vaccine adherence is a strong predictor of willingness to accept emerging vaccines. The level of correct knowledge about mpox among medical students was associated with vaccine willingness: each unit decrease in knowledge score increased hesitancy (OR 0.948, 95% CI 0.941‐0.955). This finding echoes the conclusion from a Nigerian study that “knowledge gaps lead to barriers in prevention and control” [32]. Medical students with incomplete knowledge may misunderstand transmission routes or overestimate vaccine side effects, resulting in hesitation. Within the KAP framework, these factors do not exist in isolation. Knowledge of emerging infectious diseases, as an identified influencing factor, may affect individuals’ vaccination attitudes, and such attitudes may further impact vaccination willingness. Past vaccination practices may consolidate vaccination attitudes, which is supported by our single-factor analysis results showing that previous influenza and COVID-19 vaccination history strengthened mpox vaccination willingness. These findings indicate that vaccine hesitancy may arise from the interaction of knowledge, attitudes, and practices.

Vaccine refusal was associated with 6 factors: male sex, high academic year, having relatives or friends who worked on the COVID-19 frontline, no internship experience, presence of organic diseases, and low mpox knowledge. Conversely, good self-reported health status was a protective factor. Male participants were 1.641-fold more likely to refuse (OR 1.641, 95% CI 1.003‐2.686), and each increase in academic year raised refusal risk by 44.2% (OR 1.442, 95% CI 1.154‐1.802). The sex effect is more pronounced for refusal than hesitancy, suggesting male individuals may hold more entrenched skepticism, possibly due to overconfidence in clinical knowledge or exposure to niche medical debates about vaccine use. Higher-year students, while more knowledgeable about clinical practice, may also be more exposed to anecdotal reports of vaccine adverse events, which could foster increased skepticism toward public health guidelines. This skepticism may manifest as deliberate rejection rather than indecision. Studies on American medical students also found that 25.1% of the students believed that vaccine education was insufficient, and 8.6% refused to encourage patients to receive the COVID-19 vaccine [33]. Such findings suggest that clinical training should strengthen education on “the dialectical relationship between individual cases and population data.” Participants with relatives or friends on the COVID-19 frontline had a 2.233-fold higher refusal risk (OR 2.233, 95% CI 1.337‐3.764). This counterintuitive finding may stem from unique knowledge sources and consequent attitude shifts. Their proximity to frontline workers provides them with first-hand, often stressful, knowledge regarding systemic challenges, health care worker overwork, and rare adverse events. This direct or relayed knowledge can critically shape attitudes, eroding trust in official vaccine recommendations, which is consistent with the research findings on high vaccine hesitancy toward the COVID-19 vaccine in health care workers and health care students worldwide [34]. This finding also aligns with the conclusion that pandemic fatigue among the Chinese public in the postpandemic era may hinder the conversion of vaccine uptake willingness into actual behavior [35], suggesting that authorities must rebuild the trust of professional groups by enhancing transparency in epidemic prevention and control processes. Students without internships were 2.049-fold more likely to refuse (OR 2.049, 95% CI 1.076‐3.901). Clinical internships expose students to real-world cases and reinforce vaccine use through direct observation of patient outcomes. Without this experiential learning, vaccine refusal may stem from abstract, theoretical skepticism rather than evidence-based reasoning. Individuals with organic diseases had the highest refusal risk (OR 3.733, 95% CI 1.780‐7.838). This result is clinically rational: those with chronic conditions may fear vaccine-disease interactions or adverse effects exacerbating their condition. Even with medical training, concerns about individual health vulnerability likely override general preventive benefits, highlighting the need for personalized vaccine counseling [36,37]. Concerns about vaccine safety are also reflected in surveys conducted among college students in southwest China [38]. Low knowledge increased refusal (OR 0.954, 95% CI 0.938‐0.971), while good health reduced it (OR 0.748, 95% CI 0.580‐0.965). Healthy students may perceive fewer barriers to vaccination and more benefits. By contrast, knowledge deficits amplify misconceptions [39]; for example, conflating mpox with low-prevalence diseases, leading to active rejection.

### Limitations

The following limitations should be acknowledged. First, the snowball sampling in this study was a nonprobability sample, as all participants enrolled on a voluntary basis. Therefore, the recruitment networks may have over-represented students with stronger social connections or a pre-existing interest in public health, which could introduce selection bias. Second, 4 experts in public health and health education reviewed the questionnaire for content validity, while factor analysis results were lacking. Third, self-reported vaccination willingness may be subject to social desirability bias, which may have been heightened by mandatory response items, regulated minimum response time, and the participants’ identity as medical students. Fourth, the study sample was confined to medical students, which limits the generalizability of the findings to nonmedical students or other populations. Finally, the use of a self-designed questionnaire may limit comparability with other studies.

### Conclusions

This study demonstrates high mpox vaccination acceptance among Chinese medical students. Factors associated with vaccination hesitancy included male sex, being an only child, no history of COVID-19 in family or friends, no influenza vaccination, and low mpox knowledge. Factors associated with vaccination rejection included male sex, high academic grade, family or friends working on COVID-19 frontlines, no internship experience, and presence of organic diseases. Good self-reported health status was a protective factor against refusal. The identified influencing factors provide actionable insights for policymakers, offering a reference for future risk assessment, emergency response, and management during similar public health emergencies. For medical education, the findings point to specific, operational measures: integrating mpox education into medical curricula to address knowledge gaps; designing targeted messaging for higher-risk subgroups such as male individuals and senior students; leveraging medical students as trusted community health advocates; and rebuilding trust through transparent communication, particularly among those affected by pandemic fatigue. These measures not only contribute to controlling current mpox outbreaks but also strengthen long-term public health resilience against future zoonotic disease threats.

## Supplementary material

10.2196/86981 Multimedia Appendix 1 Demographic characteristics of Chinese medical students and their willingness to receive monkeypox (mpox) vaccination.

10.2196/86981 Checklist 1 STROBE checklist.

## Abbreviations

KAP: knowledge, attitudes, and practices
mpox: monkeypox
MPXV: monkeypox virus
OR: odds ratio
SPSS: Statistical Package for the Social Sciences
STROBE: Strengthening the Reporting of Observational Studies in Epidemiology
