# Supplementary material for: Knowledge, Attitudes, Practices, and Vaccination Willingness Toward Mpox (Monkeypox) Among Chinese Medical Students: Cross-Sectional Study
Source: JMIR Public Health Surveill. 2026 Feb 6;12:e86981. doi: 10.2196/86981 (PMC12880590; doi:10.2196/86981)
Supplement: Multimedia Appendix 1 [file publichealth-v12-e86981-s001.doc]

Multimedia Appendix 1 Demographic characteristics of Chinese medical students and their willingness to receive mpox (monkeypox) vaccination.

| Variables | All participants  *n* (%) | Willingness to receive mpox vaccine, *n* (%) | | | Chi-square (*df*) | *P* value |
| --- | --- | --- | --- | --- | --- | --- |
|  |  | Acceptance | Hesitancy | Rejection |  |  |
| Total | 4098 (100) | 3468 (84.63) | 550 (13.42) | 80 (1.95) |  |  |
| Gender |  |  |  |  | 42.5 (2) | <.001^a^ |
| Male | 1739 (42.44) | 1398 (80.39) | 294 (16.91) | 47 (2.70) |  |  |
| Female | 2359 (57.56) | 2070 (87.75) | 256 (10.85) | 33 (1.40) |  |  |
| Grade |  |  |  |  | 23.8 (8) | .003^a^ |
| First | 1731 (42.24) | 1489 (86.02) | 219 (12.65) | 23 (1.33) |  |  |
| Second | 911 (22.23) | 787 (86.39) | 108 (11.86) | 16 (1.76) |  |  |
| Third | 880 (21.47) | 732 (83.18) | 121 (13.75) | 27 (3.07) |  |  |
| Fourth | 314 (7.66) | 252 (80.25) | 56 (17.83) | 6 (1.91) |  |  |
| Fifth | 262 (6.39) | 208 (79.39) | 46 (17.56) | 8 (3.05) |  |  |
| Major |  |  |  |  | 1.1 (2) | .59 |
| Clinical Medicine | 3009 (73.43) | 2539 (84.38) | 413 (13.73) | 57 (1.89) |  |  |
| Others | 1089 (26.57) | 929 (85.31) | 137 (12.58) | 23 (2.11) |  |  |
| Region |  |  |  |  | 0.9 (2) | .64 |
| Urban areas | 2365 (57.71) | 2012 (85.07) | 309 (13.07) | 44 (1.86) |  |  |
| Rural areas | 1733 (42.29) | 1456 (84.02) | 241 (13.91) | 36 (2.08) |  |  |
| Only child |  |  |  |  | 8.2 (2) | .02^a^ |
| Yes | 1727 (42.14) | 1429 (82.74) | 261 (15.11) | 37 (2.14) |  |  |
| No | 2371 (57.86) | 2039 (86.00) | 289 (12.19) | 43 (1.81) |  |  |
| Poor student identified by the school |  |  |  |  | 5.6 (2) | .06 |
| Yes | 1002 (24.45) | 829 (82.73) | 146 (14.57) | 27 (2.69) |  |  |
| No | 3096 (75.55) | 2639 (85.24) | 404 (13.05) | 53 (1.71) |  |  |
| Student leader |  |  |  |  | 0.3 (2) | .84 |
| Yes | 1043 (25.45) | 885 (84.85) | 136 (13.04) | 22 (2.11) |  |  |
| No | 3055 (74.55) | 2583 (84.55) | 414 (13.55) | 58 (1.90) |  |  |
| The reason for choosing medicine. |  |  |  |  | 11.1 (2) | .004^a^ |
| Own desire | 3153 (76.94) | 2699 (85.60) | 400 (12.69) | 54 (1.71) |  |  |
| Suggested by others | 945 (23.06) | 769 (81.38) | 150 (15.87) | 26 (2.75) |  |  |
| Do your parents or relatives have medical workers? |  |  |  |  | 4.9 (2) | .09 |
| Yes | 1540 (37.58) | 1319 (85.65) | 186 (12.08) | 35 (2.27) |  |  |
| No | 2558(62.42) | 2149 (84.01) | 364 (14.23) | 45 (1.76) |  |  |
| Do your parents or relatives engage in frontline work during the COVID-19 outbreak? |  |  |  |  | 10.0 (2) | .007^a^ |
| Yes | 822 (20.06) | 692 (84.18) | 103 (12.53) | 27 (3.28) |  |  |
| No | 3276 (79.94) | 2776 (84.74) | 447 (13.64) | 53 (1.62) |  |  |
| Do you have internship experience? |  |  |  |  | 5.6 (2) | .06 |
| Yes | 1254 (30.60) | 1039 (82.85) | 192 (15.31) | 23 (1.83) |  |  |
| No | 2844 (69.40) | 2429 (85.41) | 358 (12.59) | 57 (2.00) |  |  |
| Self-health status |  |  |  |  | 59.4 (8) | <.001^a^ |
| Very Poor | 66 (1.61) | 43 (65.15) | 14 (21.21) | 9 (13.64) |  |  |
| Poor | 209 (5.10) | 174 (83.25) | 32 (15.31) | 3 (1.44) |  |  |
| Average | 1693 (41.31) | 1414 (83.52) | 245 (14.47) | 34 (2.01) |  |  |
| Good | 1448 (35.33) | 1246 (86.05) | 178 (12.29) | 24 (1.66) |  |  |
| Very Good | 682 (16.64) | 591 (86.66) | 81 (11.88) | 10 (1.47) |  |  |
| Chronic disease |  |  |  |  | 14.1 (2) | .001^a^ |
| Yes | 329 (8.03) | 264 (80.24) | 50 (15.20) | 15 (4.56) |  |  |
| No | 3769 (91.97) | 3204 (85.01) | 500 (13.27) | 65 (1.72) |  |  |
| Allergic constitution |  |  |  |  | 8.6 (2) | .01^a^ |
| Yes | 600 (14.64) | 491 (81.83) | 89 (14.83) | 20 (3.33) |  |  |
| No | 3498 (85.36) | 2977 (85.11) | 461 (13.18) | 60 (1.72) |  |  |
| Organic disease |  |  |  |  | 26.0 (2) | <.001^a^ |
| Yes | 143 (3.49) | 111 (77.62) | 21 (14.69) | 11 (7.69) |  |  |
| No | 3955 (96.51) | 3357 (84.88) | 529 (13.38) | 69 (1.74) |  |  |
| Have you, your relatives, or friends ever had COVID-19? |  |  |  |  | 40.6 (2) | <.001^a^ |
| Yes | 3566 (87.02) | 3067 (86.01) | 434 (12.17) | 65 (1.82) |  |  |
| No | 532 (12.98) | 401 (75.38) | 116 (21.80) | 15 (2.82) |  |  |
| Have you received the influenza vaccination? |  |  |  |  | 24.2 (2) | <.001^a^ |
| Yes | 3095 (75.52) | 2668 (86.20) | 373 (12.05) | 54 (1.74) |  |  |
| No | 1003 (24.48) | 800 (79.76) | 177 (17.65) | 26 (2.59) |  |  |
| Have you received the COVID-19 vaccination? |  |  |  |  | 47.6 (2) | <.001^a^ |
| Yes | 4012 (97.90) | 3418 (85.19) | 518 (12.91) | 76 (1.89) |  |  |
| No | 86 (2.10) | 50 (58.14) | 32 (37.21) | 4 (4.65) |  |  |
| The reason for receiving COVID-19 vaccination. |  |  |  |  | 35.4 (2) | <.001^a^ |
| Own desire | 3048 (75.97) | 2648 (86.88) | 358 (11.75) | 42 (1.38) |  |  |
| Suggested by others | 964 (24.03) | 770 (79.88) | 160 (16.60) | 34 (3.53) |  |  |
| Were you placed under centralized quarantine due to the COVID-19 pandemic? |  |  |  |  | 0.9 (2) | .63 |
| Yes | 1244 (30.36) | 1060 (85.21) | 158 (12.70) | 26 (2.09) |  |  |
| No | 2854 (69.64) | 2408 (84.37) | 392 (13.74) | 54 (1.89) |  |  |

^a^ *P*<.05 and the difference were statistically significant.
